# Transcriptome analysis reveals the mechanism of stromal cell-derived factor-1 and exendin-4 synergistically promoted periodontal ligament stem cells osteogenic differentiation

**DOI:** 10.7717/peerj.12091

**Published:** 2021-08-27

**Authors:** Wenyan Kang, Lingqian Du, Qianyu Liang, Rui Zhang, Chunxu Lv, Shaohua Ge

**Affiliations:** 1 Department of Periodontology, School and Hospital of Stomatology, Cheeloo College of Medicine, Shandong University & Shandong Key Laboratory of Oral Tissue Regeneration & Shandong Engineering Laboratory for Dental Materials and Oral Tissue Regeneration, Jinan, Shandong, China; 2 Department of Stomatology, The Second Hospital, Cheeloo College of Medicine, Shandong University, Jinan, Shandong, China

**Keywords:** Periodontal ligament stem cells, Stromal cell-derived factor-1, Exendin-4, Osteogenic differentiation, RNA-seq

## Abstract

Stromal cell-derived factor-1 (SDF-1) and Exendin-4 (EX-4) play beneficial roles in promoting periodontal ligament stem cells (PDLSCs) osteogenic differentiation, while the detailed mechanism has not been clarified. In this study, we aimed to evaluate the biological mechanism of SDF-1 and EX-4 alone or synergistic application in regulating PDLSCs differentiation by RNA-sequencing (RNA-seq). A total of 110, 116 and 109 differentially expressed genes (DEGs) were generated in osteogenic medium induced PDLSCs treated by SDF-1, EX-4, and SDF-1+EX-4, respectively. The DEGs in SDF-1 group were enriched in signal transduction related signaling pathways; the DEGs in EX-4 group were enriched in metabolism and biosynthesis-related pathways; and the DEGs generated in SDF-1+EX-4 group were mainly enriched in RNA polymerase II transcription, cell differentiation, chromatin organization, protein phosphorylation pathways. Based on Venn analysis, a total of 37 specific DEGs were identified in SDF-1+EX-4 group, which were mainly enriched in negative regulation of autophagy and cellular component disassembly signaling pathways. Short time-series expression miner (STEM) analysis grouped all expressed genes of PDLSCs into 49 clusters according to the dynamic expression patterns and 25 genes, including NRSN2, CHD9, TUBA1A, distributed in 10 gene clusters in SDF-1+EX-4 treated PDLSCs were significantly up-regulated compared with the SDF-1 and EX-4 alone groups. The gene set enrichment analysis indicated that SDF-1 could amplify the role of EX-4 in regulating varied signaling pathways, such as type II diabetes mellitus and insulin signaling pathways; while EX-4 could aggravate the effect of SDF-1 on PDLSCs biological roles *via* regulating primary immunodeficiency, tight junction signaling pathways. In summary, our study confirmed that SDF-1 and EX-4 combined application could enhance PDLSCs biological activity and promote PDLSCs osteogenic differentiation by regulating the metabolism, biosynthesis and immune-related signaling pathways.

## Introduction

Periodontal disease-induced tooth loss has become a global public health challenge that greatly affects people’s quality of life (Peres et al., 2019). Mesenchymal stem cell (MSC) based periodontal tissue regeneration has aroused great attention in the field of regenerative medicine (Hu, Liu & Wang, 2018). Among all MSCs, periodontal ligament stem cells (PDLSCs) are the main candidate cells for periodontal regeneration. Several studies have demonstrated that transplanting autologous and allogeneic PDLSCs directly into periodontal defect areas or surgically created periodontal defect areas could result in periodontal tissue regeneration, which highlights that PDLSC-mediated tissue engineering is a promising therapy for periodontitis (Bartold, Shi & Gronthos, 2006; Ding et al., 2010; Liu et al., 2008; Liu et al., 2019a; Liu et al., 2019b).

An increasing number of researchers have focused on the recruitment of endogenous PDLSCs to the injury site to enhance healing by harnessing the innate regenerative potential of the body (Lee et al., 2017a). Cytokines, chemokines, and adhesion molecules have been used to enhance cell migration, maintain tissue homeostasis, regulate immune responses, promote wound healing and facilitate periodontal tissue regeneration (Lee et al., 2017b; Onizuka & Iwata, 2019; Wang et al., 2013). Stromal cell-derived factor-1 (SDF-1), a member of the chemokine family, can promote the proliferation and migration of various MSCs by activating the G protein-coupled receptor C-X-C chemokine receptor type 4 (CXCR4) (Du, Feng & Ge, 2016; Kimura et al., 2014; Zhu, Dissanayaka & Zhang, 2019). Our previous study also demonstrated that topical application of SDF-1 could significantly recruit MSCs to the wound area and promote local vascular regeneration in a rat model (Wang, Du & Ge, 2016). SDF-1 possesses great potential in promoting MSC migration and growth; however, the compromised osteogenic differentiation of these cells could not be induced by SDF-1. Therefore, the application of SDF-1 alone is insufficient for favorable bone regeneration, and the optimal method for potentiating periodontal bone regeneration is to combine SDF-1 with other osteogenic factors.

Exendin-4 (EX-4), a full agonist of glucagon-like peptide-1 receptor (GLP-1R), has been widely used in the clinical treatment of type 2 diabetes mellitus (T2DM) (Yap & Misuan, 2019). In addition, EX-4 plays key roles in promoting MSC proliferation and migration (Zhang et al., 2016; Zhou et al., 2015a). Recently, EX-4 has been confirmed to present the potential to promote osteogenic differentiation and bone formation in a variety of stem/precursor cells (Feng et al., 2016; Luciani et al., 2018; Meng et al., 2016). Moreover, in addition to enhancing the MSC osteogenic differentiation capability, EX-4 could promote the recruitment effect of SDF-1 (Zhou et al., 2015b). Our previous study also confirmed that SDF-1 and EX-4 cotherapy synergistically promoted PDLSCs proliferation, migration and osteogenic differentiation (Liang et al., 2021). However, the mechanism of SDF-1 and EX-4 alone or synergetic application for PDLSCs osteogenic differentiation is not fully understood.

High-throughput RNA sequencing (RNA-Seq) has been widely applied to analyze the whole transcriptomic changes of eukaryotes, which can provide progressively greater knowledge of both the quantitative and qualitative aspects of transcript biology (Ozsolak & Milos, 2011). RNA-seq has been successfully applied to identify the potential transcriptional mechanisms of various diseases, such as cancers, metabolic diseases and retinal diseases (Bakhtiarizadeh et al., 2019; Demircioğlu et al., 2019; Farkas et al., 2015). In the present study, RNA-seq transcriptomic analysis was applied to identify the core dynamic differentially expressed genes (DEGs) signature affected by EX-4 and SDF-1 alone or synergistically application in osteogenic medium-induced PDLSCs. Additionally, an integrated network containing specific DEGs generated in EX-4+SDF-1-treated PDLSCs was constructed. The results revealed the whole alteration of gene expression in PDLSCs undergoing EX-4 and SDF-1 application during the osteogenic differentiation process, which establishes a foundation for further research investigating the synergistic application of SDF-1 and EX-4 to promote PDLSCs osteogenic differentiation.

## Materials and Methods

### Human subjects and ethics statements

This study was approved by the Medical Ethical Committee of the Stomatology School, Shandong University (NO. 20170801). Five healthy individuals without systemic diseases aged from 18–30 who underwent premolar extraction at the Department of Oral and Maxillofacial Surgery were recruited for this project. All individuals agreed to participate in the research project and signed the informed consent forms according to the Helsinki Declaration of 1975.

### Cell isolation and culture

The extracted teeth were stored in Dulbecco’s modified Eagle’s medium (DMEM, HyClone, Logan, UT, USA) with 5% antibiotics (100 U/mL penicillin, 100 mg/mL streptomycin, Sigma Aldrich, St Louis, MO, USA) and quickly transported from the clinic to the laboratory. Then, single PDLSCs were acquired as previously described in our previous study (Du, Feng & Ge, 2016). Specifically, Primary PDLSCs were cultured with DMEM containing 20% fetal bovine serum (FBS, BioInd, Kibbutz, Israel) at 37 °C in a humidified atmosphere of 5% CO_2_, and cells were trypsinized and passaged at a dilution ratio of 1:3 to expand the culture in 10% FBS medium upon the cell monolayer reached 80–90% confluence. Fourth passage cells were used in all experiments.

### RNA-seq analysis

PDLSCs from 5 different individuals were cultured in osteogenic medium (OM) and treated with SDF-1, EX-4 or SDF-1+EX-4 at 21 d, and normal PDLSCs treated with OM served as a negative control (NC). Totally, we collected 20 samples and RNA-seq was used to analyze the whole genome expression at LC Sciences through the Illumina X10 platform (Hangzhou, Zhejiang, China). Firstly, the total RNA were performed quality control based on previous study (Wang, Wang & Li, 2012), and then the clean reads were mapped to the reference genome (GRCh38) *via* hierarchical indexing for spliced alignment of transcripts (HISAT) (v2.0.4) (Kim, Langmead & Salzberg, 2015). The mapped reads of each sample were assembled using StringTie (v1.3.0, Pertea et al., 2015). Furthermore, all transcriptomes samples were merged to reconstruct a comprehensive transcriptome using Perl scripts. After the final transcriptome was generated, StringTie and edgeR were used to estimate the expression levels of all transcripts. StringTie was used to determine the expression level of mRNAs by calculating fragments per kilobase of exon model per million mapped fragments (FPKM). The DEGs were selected with statistical significance (*p* value <0.05) by R package (v 3.2.5).

### Volcano analysis of DEGs in PDLSCs

Volcano analysis was used to identify the DEGs between each pair of groups (Li, 2012). The up- and down-regulated genes were identified, and the total number of each pair of groups was visualized by the histogram.

### Gene ontology (GO) and kyoto encyclopedia of genes and genomes (KEGG) enrichment analysis

Based on the DEGs generated by SDF-1, EX-4 and SDF-1+EX-4 compared with NC, the overrepresented GO categories and the significant KEGG pathways were identified (anonymous, 2019; Kanehisa & Goto, 2000). A *q* value <0.05 was used as the cut-off criterion for the selection of significant GO terms and KEGG pathways.

### Venn and Upset analysis

To identify the specific DEGs generated by every two different compared groups, overlapping analysis was performed according to the jvenn website (http://jvenn.toulouse.inra.fr/app/example.html), and an intersection UpSet diagram based on the UpSet R package was drawn (Conway, Lex & Gehlenborg, 2017). The specific genes generated by each group were identified and the gene functions were analyzed according to Metascape website (http://metascape.org/gp/index.html#/main/step1) (Zhou et al., 2019).

### Short time-series expression miner (STEM) analysis

STEM software (version 1.3.8) (Douglas et al., 2019) was applied to identify the specific gene expression clusters in PDLSCs treated with SDF-1, EX-4 and SDF-1+EX-4. The genes in the upregulated clusters in SDF-1+EX4-treated PDLSCs were selected, and the expression of all genes in these clusters was shown by a heatmap. The interaction relationship of these genes was analyzed according to the GeneMANIA database (http://genemania.org/search) (Franz et al., 2018).

### In-depth mechanism analysis and functional network construction

To identify the function of specific DEGs generated by SDF-1, EX-4 and SDF-1+EX-4-treated PDLSCs, a functional network was constructed according to STRING database (https://string-db.org/) and GeneMANIA database (http://genemania.org/search) (Franz et al., 2018). Functional enrichment analysis of genes in the functional network was further performed by the Metascape database (Zhou et al., 2019).

### Gene set enrichment analysis (GSEA)

GSEA is one of the functional class scoring analysis methods (Subramanian et al., 2005). To select the genes that were not significantly differentially expressed but were important for the function of biological pathways, GSEA was performed method according to all genes in SDF-1 *vs* NC, EX-4 *vs* NC, SDF-1+EX-4 *vs* NC, SDF-1+EX-4 *vs* EX-4, SDF-1+EX-4 *vs* SDF-1, and EX-4 *vs* SDF-1 groups according to the clusterProfiler and enrichplot R package.

## Results

### DEG analysis

**Figure 1:**
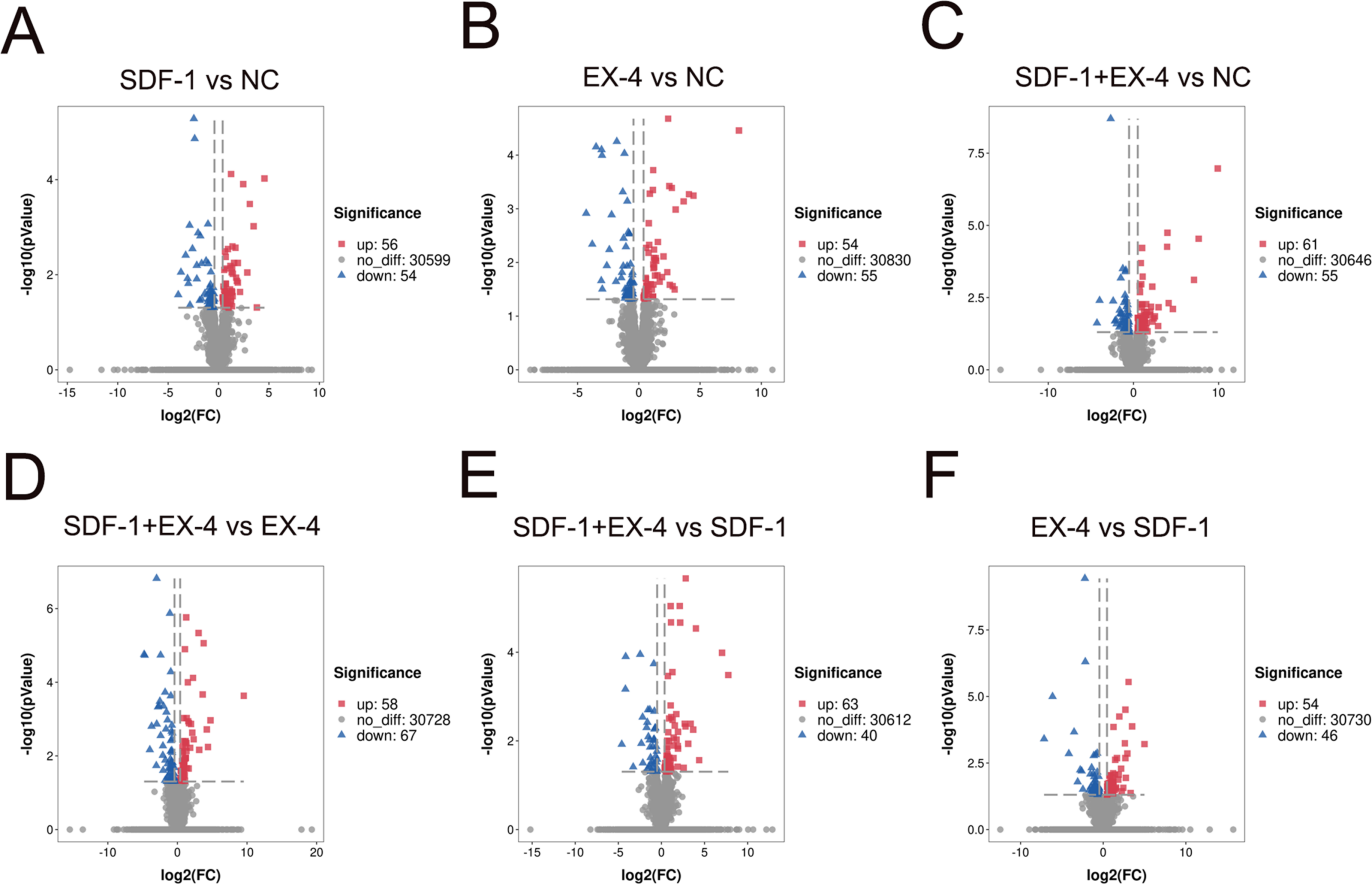
Volcano diagram of differentially expressed genes (DEGs) in PDLSCs. The DEGs in SDF-1 *vs* NC (A), EX-4 *vs* NC (B), SDF-1+EX-4 *vs* NC(C), SDF-1+EX-4 *vs* EX-4 (D), SDF-1+EX-4 *vs* SDF-1 (E), and EX-4 *vs* SDF-1 (F). Each point represents individual genes. Black dots represent the genes that were not significantly differentially expressed, red dots indicate the genes that were significantly upregulated and blue dots indicate the genes that were downregulated (—FC— > 1 and *p*-adjusted value < 0.05). The volcano plots were analyzed by DESeq2.

To investigate changes in gene expression profiles in SDF-1, EX-4 or SDF-1+EX-4- treated PDLSCs, FPKM expression values of the genes were calculated based on the read counts using featureCounts software. The fold change (FC) values of each gene at different time points post stimulation compared with NC were also calculated using DESeq2 R package. The thresh old value of —FC—>1 and FDR ≤ 0.05 was used to identify DEGs between two different groups, and the results indicated that 110, 116, 109, 125, 103 and 100 DEGs were generated in different comparison groups SDF-1 *vs* NC, EX-4 *vs* NC, SDF-1+EX-4 *vs* NC, SDF-1+EX-4 *vs* EX-4, SDF-1+EX-4 *vs* SDF-1, EX-4 *vs* SDF-1 (Fig. 1). Among all compared DEGs, 56, 61, 54, 58, 63 and 54 upregulated genes and 54, 55, 55, 67, 40 and 46 downregulated genes were identified (Fig. S1).

### GO and KEGG enrichment analysis

**Figure 2:**
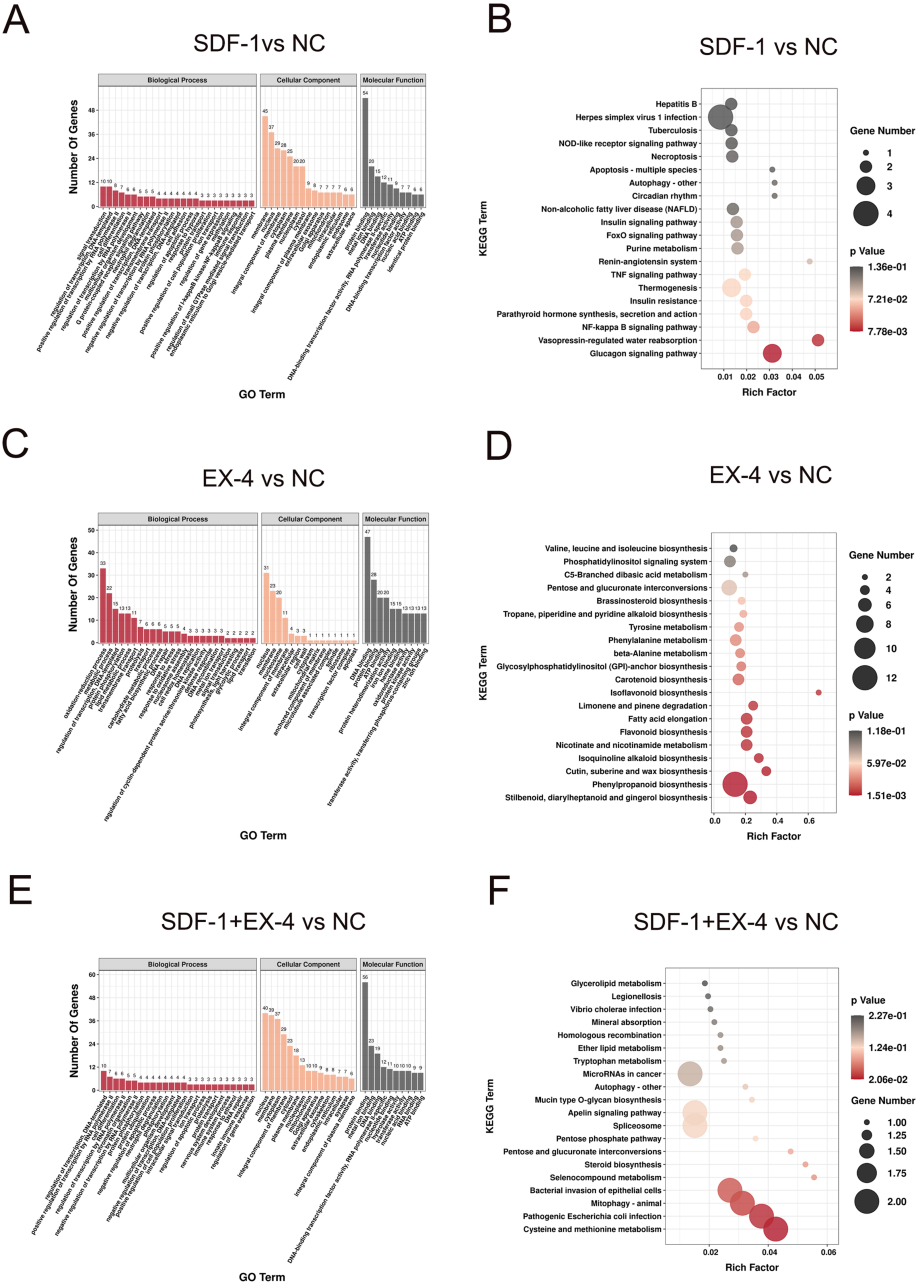
Functional enrichment analysis of DEGs in compared groups. (A) GO enrichment analysis of the DEGs in SDF-1 *vs* NC. (B) KEGG enrichment analysis of the DEGs in SDF-1 *vs* NC. (C) GO enrichment analysis of the DEGs in EX-4 *vs* NC. (D) KEGG enrichment analysis of the DEGs in EX-4 *vs* NC. (E) GO enrichment analysis of the DEGs in SDF-1+EX-4 *vs* NC. (F) KEGG enrichment analysis of the DEGs in SDF-1+EX-4 *vs* NC. Red bar represents the enriched signaling pathways according to the biological process. Orange bar represents the enriched signaling pathways according to the cellular component. Gray bar represents the enriched signaling pathways according to the molecular function (A, C, E). The size of the symbol represents the gene counts enriched in the signaling pathways. The color indicates the degree of statistical significance (B, D, F).

The GO (biological process) enrichment results showed that the DEGs generated by SDF-1 stimulation were mainly enriched in signal transduction regulation of transcription, cell differentiation, RNA polymerase II transcription, and multicellular organism development signaling pathways (Fig. 2A and Table S1). The KEGG analysis results also indicated that SDF-1-induced DEGs were mainly enriched in the glucagon signaling pathway, NF-kappa B signaling pathway, and TNF signaling pathway (Fig. 2B and Table S1), and mostly involved in human diseases and organismal systems, such as viral infectious diseases, the immune system, and the endocrine system (Fig. S2B and Table S1). In addition, the DEGs generated by the EX-4 stimulation were mainly enriched in the oxidation–reduction process, metabolic process, regulation of transcription, DNA-templated, protein phosphorylation, and lipid metabolic process pathways (Fig. 2C and Table S1). KEGG analysis also indicated that EX-4-induced DEGs were mainly enriched in metabolism and biosynthesis-related pathways, such as nicotinate and nicotinamide metabolism, phenylpropanoid biosynthesis, and phenylpropanoid biosynthesis (Fig. 2D and Table S1), and all these DEGs were significantly involved in energy metabolism, lipid metabolism and biosynthesis of other secondary metabolites (Fig. S2D). Moreover, we identified that the DEGs generated by SDF-1+EX-4 was also mainly enriched in RNA polymerase II transcription, cell differentiation, chromatin organization, and protein phosphorylation pathways according to the GO (biological process) analysis results (Fig. 2E and Table S1). The KEGG enrichment analysis results indicated that these costimulated DEGs were mainly enriched in pathogenic Escherichia coli infection, mitophagy in animals, bacterial invasion of epithelial cells and cysteine and methionine metabolism signaling pathways (Fig. 2F and Table S1). Additionally, we found that all the DEGs generated by the SDF-1, EX-4, SDF-1+EX-4 groups were significantly enriched in the nucleus, membrane, nucleosome and cytoplasm pathways according to the GO cellular component analysis, and based on the GO molecular function analysis, all these DEGs were involved in the DNA binding, protein binding, ATP binding and protein heterodimerization activity pathways (Figs. 2A, 2C, 2E and Figs. S2A, S2C, S2E).

### Screening of specific DEGs and functional analysis

**Figure 3:**
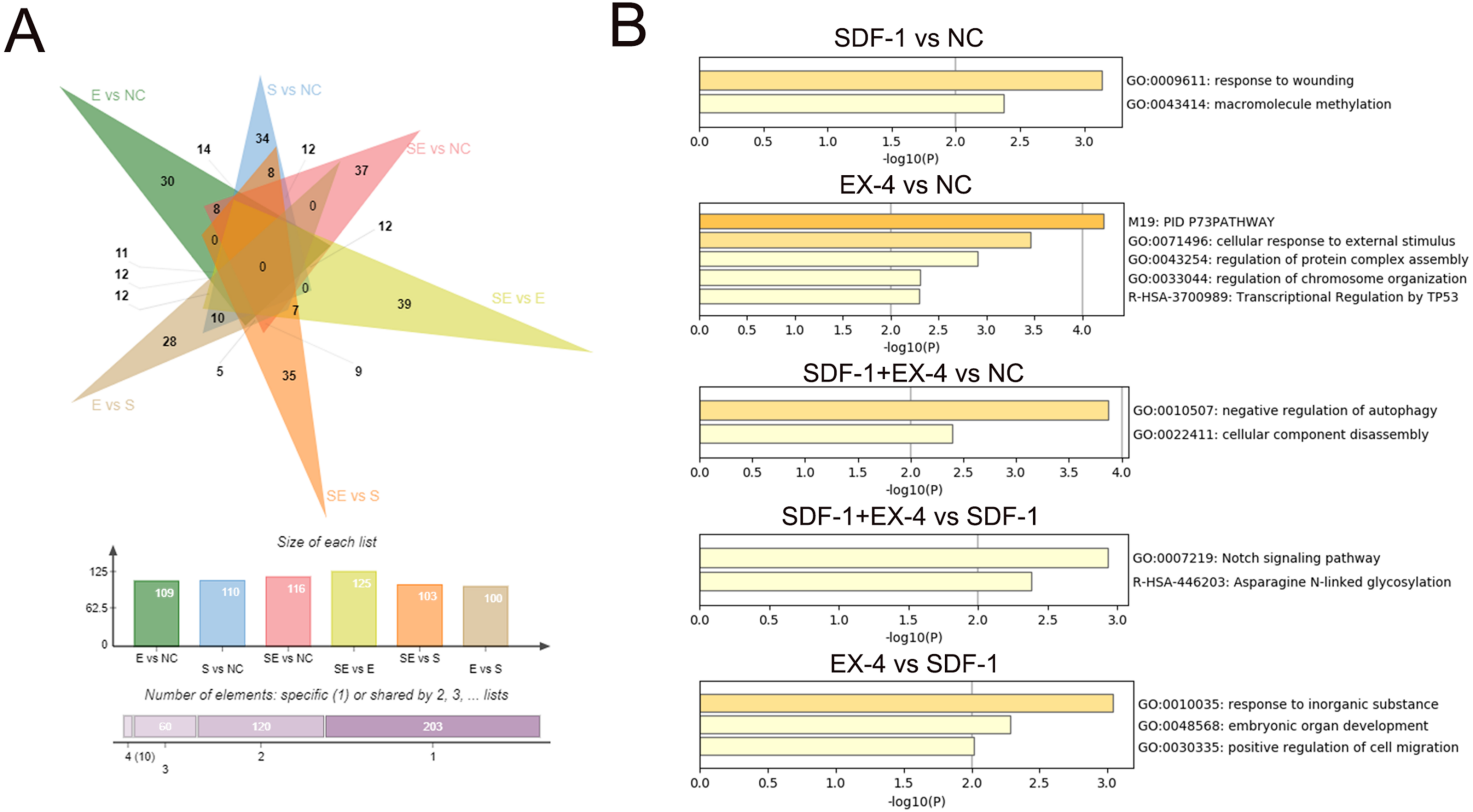
Screening of specific DEGs and function analysis of each compared group. (A) Venn analysis of the DEGs among SDF-1 *vs* NC, EX-4 *vs* NC, SDF-1+EX-4 *vs* NC, SDF-1+EX-4 *vs* EX-4, SDF-1+EX-4 *vs* SDF-1, and EX-4 *vs* SDF-1. (B) Functional enrichment analysis of the specific DEGs in each two compared groups (SDF-1 *vs* NC, EX-4 *vs* NC, SDF-1+EX-4 *vs* NC, SDF-1+EX-4 *vs* SDF-1, EX-4 *vs* SDF-1), which were exhibited in the outermost layer in the Venn analysis.

To characterize the DEGs specifically generated by SDF-1 *vs* NC, EX-4 *vs* NC, SDF-1+EX-4 *vs* NC, SDF-1+EX-4 *vs* EX-4, SDF-1+EX-4 *vs* SDF-1, and EX-4 *vs* SDF-1, we performed an overlapped analysis of the six compared DEG groups, and the results indicated that 34, 30, 37, 39, 35 and 28 DEGs were specially generated (Fig. 3A, Table S2, Fig. S3 and Table S3). In addition, the Metascape website was referenced to analyze the specific gene functions in different groups and the results indicated that the 34 specific DEGs generated in SDF-1 *vs* NC was mainly enriched in response to the wounding, and macromolecule methylation signaling pathways; the 30 specific DEGs generated in EX-4 *vs* NC were mainly enriched in the cellular response to external stimulus, regulation of protein complex assembly, and regulation of chromosome organization signaling pathways; the 37 specific DEGs generated in SDF-1+EX-4 *vs* NC were mainly enriched in the negative regulation of autophagy and cellular component disassembly signaling pathways; the 39 specific DEGs generated in SDF-1+EX-4 *vs* SDF-1 were mainly enriched in the Notch signaling pathway and Asparagine N-linked glycosylation signaling pathways; and the 28 specific DEGs generated by EX-4 *vs* SDF-1 group were mainly enriched in the response to inorganic substance, embryonic organ development and A positive regulation of cell migration signaling pathways (Fig. 3B and Table S4).

### Screening of core DEGs generated by the SDF-1 and EX-4 combined application

**Figure 4:**
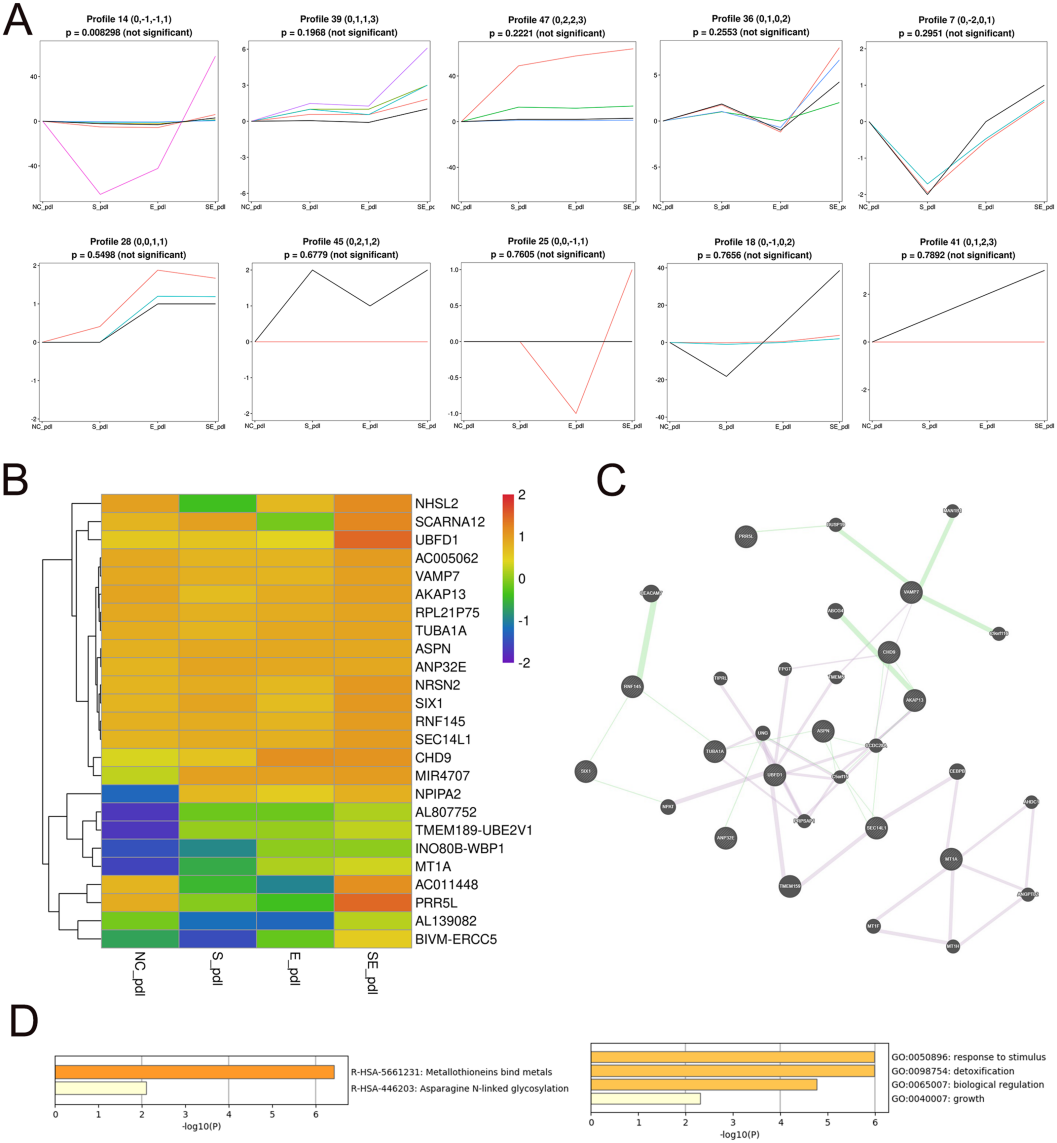
Screening of core DEGs generated by the combined SDF-1 and EX-4 application. (A) Short Time-series Expression Miner (STEM) analysis in PDLSCs cocultured with SDF-1, EX-4 and SDF-1+EX-4. Gene clusters (including 14, 39, 47, 36, 7, 28, 45, 25, 18, 41) that were upregulated by the SDF-1and EX-4 combined application were selected out (mock infection was designated as NC, the first node was SDF-1 stimulated group, the second node was EX-4 stimulated group, and the final node was SDF-1+EX-4 combined stimulated group). (B) Heat map of the 25 upregulated DEGs generated by the combined SDF-1 and EX-4 application. (C) GeneMANIA analysis network based on these 25 DEGs; the genes in circles with a white slash are the actual DEGs, and the genes in circles without a slash are genes predicated based on physical interactions, coexpression, predications, colocalization, pathways, genetic interactions and shared protein domains. (D) Functional enrichment analysis of genes presented in the GeneMANIA network of C.

To identify the core DEGs generated by the SDF-1 and EX-4 combined application, STEM software was applied to perform a pattern analysis (mock infection was designated NC), and the results revealed 49 gene clusters among all DEGs generated by SDF-1, EX-4 and SDF-1+EX-4-treated PDLSCs (Fig. S4 and Table S5). In all gene clusters, we focused on genes in clusters 14, 39, 47, 36, 7, 28, 45, 25, 18, and 41, which were significantly upregulated by the SDF-1 and EX-4 combined application compared with SDF-1 or EX-4 alone (Fig. 4A). Among the 10 clusters, a total of 25 genes, including NRSN2, CHD9, TUBA1A, AKAP13, VAMP7, NPIPA2 etc., were identified, and all DEG expression in different groups is shown by a Heatmap (Fig. 4B and Table S6). The GeneMANIA analysis network showed that these genes possessed an intricate tangle of connections through genetic interactions and co-expression pattern (Fig. 4C and Table S7). Functional enrichment analysis indicated that our core DEGs and their related genes constructed in the network were mainly enriched in the metallothioneins binding metals, asparagine N-liked glycosylation, response to stimulus, detoxification, biological regulation and growth signaling pathways (Fig. 4D and Table S8).

### Network construction and the mechanism analysis of the SDF-1 and EX-4 synergistic effect in PDLSCs

**Figure 5:**
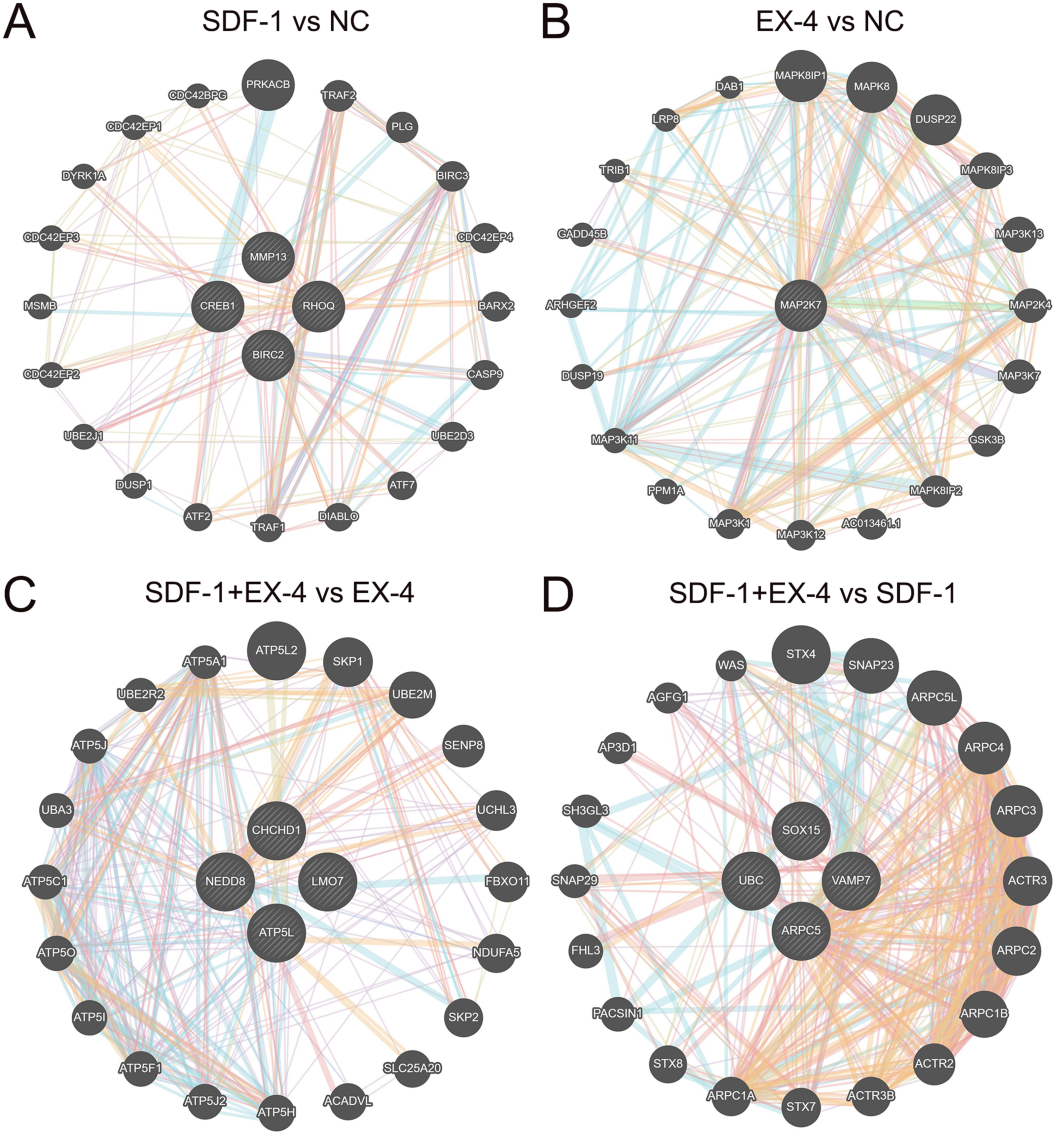
Network construction and the mechanism analysis of SDF-1 and EX-4 synergistic effects in PDLSCs. The network constructed through the GeneMANIA database based on the core DEGs in SDF-1 *vs* NC (A), EX-4 *vs* NC (B), SDF-1+EX-4 *vs* EX-4 (C), and SDF-1+EX-4 *vs* SDF-1 (D). The genes in circles with a white slash are the actual dynamic DEGs. The dynamic DEGs and predicted genes interact based on physical interactions, coexpression, predictions, colocalization, pathways, genetic interactions and shared protein domains.

To further clarify the mechanism of the SDF-1 and EX-4 combined application to promote PDLSC osteogenic differentiation, a detailed complex network analysis was performed based on the DEGs generated by the different groups through the STRING and GeneMANIA. A network analysis based on the STRING database showed that the DEGs generated by EX-4 and SDF-1 alone were centralized and converged into a network, while the DEGs in the SDF-1+EX-4 group were scattered in the network. The DEGs generated in SDF-1+EX-4 *vs* SDF-1, and SDF-1+EX-4 *vs* EX-4 were also centralized (Fig. S5). In addition, compared with the NC group, EX-4 significantly elevated the expression of the core gene MAPK27, which plays central roles by activating MAPK-related signaling pathways; and SDF-1 mainly changed the expression of the key genes CREB1, MMP13, RHOQ and BIRC2, which exert their roles by activating ATP signaling pathways (Figs. 5A and 5B). More importantly, we identified that the gene expression levels of NEDD8, CHCHD1, LMO7, and ATP5L were significantly varied the SDF-1+EX-4 *vs* EX-4 groups, which played crucial roles in activating ATP-related signaling pathways and indicated that SDF-1 could amplify the EX-4 effect in elevating ATPase activity and promoting PDLSC osteogenic differentiation. Additionally, we found that the DEGs of SOX15, UBC, VAMP7 and ARPC5 were significantly changed in SDF-1+EX-4 *vs* SDF-1 groups, which played vital roles in promoting cytoskeletal protein formation and degradation (Figs. 5C and 5D). Although the DEGs generated in the SDF-1+EX-4 *vs* NC group were dispersed, most of these genes could be centralized by the genes HAP1, TP53, TAL1, RRPF40A and TALDO1 (Fig. S6).

### Gene set enrichment analysis (GSEA) based on all genes

**Figure 6:**
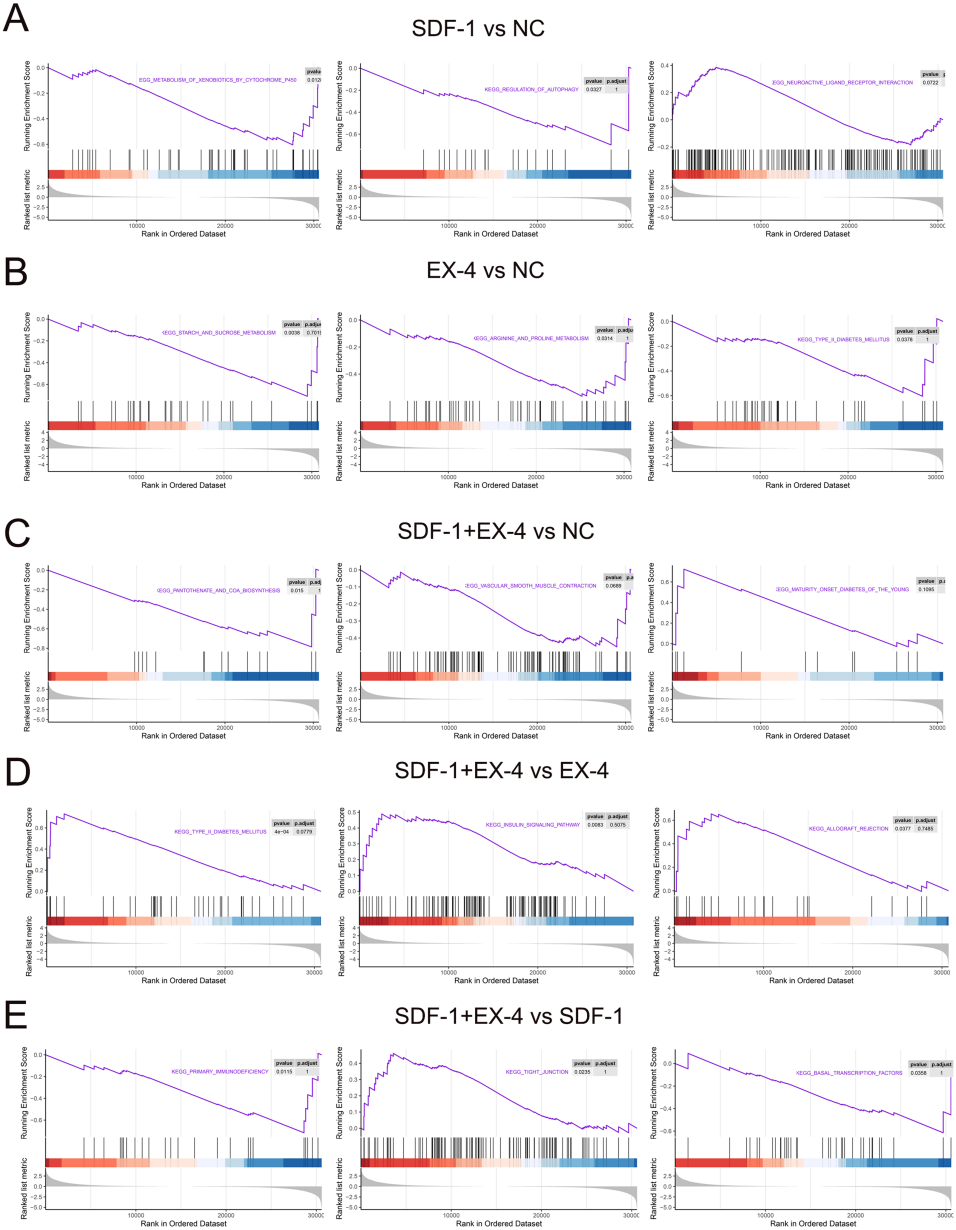
GSEA enrichment based on all genes. GSEA enrichment plots of gene expression signatures of SDF-1 *vs* NC (A), EX-4 *vs* NC (B), SDF-1+EX-4 *vs* NC (C), SDF-1+EX-4 *vs* EX-4 (D), and SDF-1+EX-4 *vs* SDF-1(E), which are sorted according to the differences between the means of samples with high and low HOTAIR expression. The barcode plot indicates the position of the genes in each gene set; red and blue colors represent positive and negative Pearson correlations with HOTAIR expression, respectively.

All of the above analyses were based on the DEGs selected according to our threshold value and statistical analysis technique; however, genes that are not significantly differentially expressed but are important for the function of the SDF-1 and EX-4 biological pathways may be omitted, and thus, we performed a GSEA according to all genes in SDF-1 *vs* NC, EX-4 *vs* NC, SDF-1+EX-4 *vs* NC, SDF-1+EX-4 *vs* EX-4, SDF-1+EX-4 *vs* SDF-1, and EX-4 *vs* SDF-1. The results indicated that SDF-1 played its roles in regulating the metabolism of xenobiotics by cytochrome P450, regulation of autophagy, neuroactive ligand receptor interaction signaling pathways (Fig. 6A and Table S9); EX-4 played roles mainly through affecting the metabolism related signaling pathways, such as, starch and sucrose metabolism, arginine and proline metabolism, and type II diabetes mellitus signaling pathways (Fig. 6B and Table S9); and the combination of SDF-1 and EX-4 mainly regulating PLDSCs biological process through activating metabolism and immunity related signaling pathways, such as pantothenate and COA biosynthesis, vascular smooth muscle contraction, and maturity onset diabetes of the young (Fig. 6C and Table S9). In addition, we confirmed that SDF-1 could amplify the role of EX-4 in regulating various signaling pathways, such as type II diabetes mellitus, the insulin signaling pathway, and the allograft rejection pathway (Fig. 6D and Table S9), while EX-4 could aggravate the effect of SDF-1 on PDLSC biological roles by regulating signaling pathways, including primary immunodeficiency, tight junction, and basal transcription factors (Fig. 6E and Table S9). Interestingly, we found that the signaling enrichment analysis based on the DEGs and GSEA shared similarity, which indicated that SDF-1 and EX-4 may regulate PDLSC biological activity by enhancing each other’s biological functions.

## Discussion

The effects of SDF-1 and EX-4 alone on bone regeneration have been widely reported; however, the synergetic effects of SDF-1 and EX-4 on PDLSC osteogenic differentiation and the potential mechanism have not been reported. In our previous study, we confirmed that the combined application of SDF-1 and EX-4 could significantly promote osteogenic differentiation of PDLSCs (Liang et al., 2021). In this study, we confirmed that SDF-1 could amplify the role of EX-4 by significantly activating metabolism-related signaling pathways, such as type II diabetes mellitus and insulin signaling pathways; and EX-4 could aggravate the effect of SDF-1 on PDLSCs biological roles by regulating primary immunodeficiency and tight junction signaling pathways. Briefly, our study confirmed that the SDF-1 and EX-4 combined application could enhance PDLSCs biological activity and promote PDLSCs osteogenic differentiation by regulating the metabolism, biosynthesis and immune-related signaling pathways.

EX-4, a common glucagon-like peptide-1 receptor agonist, has been confirmed to possess excellent effects on treating patients with T2DM by significantly reducing HbA1c content compared to basal insulins (Singh et al., 2017). Our current study confirmed that EX-4-generated DEGs were enriched in the type II diabetes mellitus signaling, starch and sucrose metabolism, arginine and proline metabolism, and alanine aspartate and glutamate metabolism signaling pathways, while the combination of SDF-1 and EX-4 could significantly activate more metabolism-related signaling pathways, such as the valine leucine and isoleucine degradation, insulin signaling, phenylalanine metabolism, and pyruvate metabolism signaling pathways. In the plasma of type II diabetes, a pronounced postprandial rise in amino acids (such as leucine, isoleucine, valine, lysine, and threonine) and glucose-dependent insulinotrophic polypeptide was observed, which finally resulted in glycemic and insulinemic responses (Nilsson, Holst & Björck, 2007). In our study, we confirmed that SDF-1 and EX-4 combination therapy could significantly increase the gene expression of EHHADH, HMGCL, IL4I1, PKLR, PIK3CG, PYGM, SLC2A4, RHOQ, PRKCZ, FBP1, and SH2B2, which play key roles in promoting amino acid degradation and insulin secretion; thus, SDF-1 assists EX-4 in controlling the blood glucose level of diabetes patients. EX-4 could promote the osteogenic differentiation of osteoblasts, adipose-derived stem cells, and PDLSCs by activating the Hedgehog/Gli1, Wnt and NF- *κ*B signaling pathways; however, the interactive relationship of EX-4 regulating PDLSCs metabolism-related pathways and osteogenic differentiation pathways and the mechanism by which SDF-1 amplifies the effect of EX-4 on PDLSCs osteogenic differentiation need to be further clarified (Deng et al., 2019; Gao, Li & Li, 2018; Liu et al., 2019a; Liu et al., 2019b).

SDF-1 could promote PDLSCs proliferation, migration and osteogenic differentiation *in vitro*, play key roles in recruiting endogenic PDLSCs into the periodontal defect area and then contribute to angiogenesis *in vivo* (Bae et al., 2017; Du, Feng & Ge, 2016). In the current study, we confirmed that SDF-1 significantly activated metabolism-related signaling pathway of PDLSCs cultured in OM, such as the metabolism of xenobiotics by cytochrome P450, alanine aspartate and glutamate metabolism; however, the effects of metabolism on PDLSCs osteogenic differentiation have not been reported. In addition, SDF-1 activated the renin angiotensin system, basal transcription factor, and neuroactive ligand receptor interaction signaling pathways, which is consistent with previous studies (Chu et al., 2009; Wang et al., 2015). Moreover, we found that the EX-4 and SDF-1 combined stimulation significantly activated the immunodeficiency, tight junction, complement and coagulation cascade signaling pathways compared to SDF-1 stimulation alone in osteogenic medium cultured PDLSCs. The adaptive immune system plays a prominent role in the development of heterotopic ossification (Ranganathan et al., 2016), and tight junctions (TJs) play a pivotal role in the modulation of paracellular permeability. For example, Cldn11 recombinant protein, a well-established component of TJs, could significantly decrease the resorption of lipopolysaccharide-induced calvarial bone and increase the osteogenic activity of calvarial bone formation (Baek et al., 2018). Moreover, stress-induced hematopoietic stem cell mobilization is enhanced by the fibrinolytic and complement cascades (Nguyen, Lapidot & Ruf, 2018). In the current study, we confirmed that EX-4 could enhance the role of SDF-1 in PDLSCs osteogenic differentiation through RNA-seq analysis, although this process was limited due to the lack of biological experimental validation. In the future, we will design related biological experiments to validate the mechanism of the SDF-1 and EX-4 combined application in promoting PDLSCs osteogenic differentiation.

In adipose-derived stem cells, EX-4 could activate the PI3K/AKT pathways and then augment the SDF-1α/CXCR4 cascade, which finally promotes cell migration (Zhou et al., 2015b). In endothelial progenitor cells, EX-4 could activate the SDF-1β/CXCR7-AMPK/p38-MAPK axis and then ameliorate high glucose-induced EPC dysfunction (Yang et al., 2020). In the current study, we confirmed that EX-4 and SDF-1 coordinate to change PDLSCs gene expression of CHD9, MT1A, RNF145, ASPN, SIX1, TUBA1A, PRR5L, UBFD1, SEC14L1, ANP32E, AKAP13, and VAMP7, which could combine with TMEM159, CEACAM7, CEBPB, ABCG4, NPAT, UNG, TMEM256, MT1F, CCDC28A, DUSP19, ANGPTL2, PRPSAP1, C9orf116, MT1H, TIPRL, MAN1B1, FPGT, AHDC1, TMEM5, and C5orf15, and then activate various signaling pathways including response to stimulus, detoxification, biological regulation and growth pathways. However, the role of these DEGs and signaling pathways identified by RNA-seq in the current study on PDLSCs osteogenic differentiation needs to be further validated.

## Conclusion

Our study confirmed that SDF-1 could amplify the role of EX-4 by activating more metabolism-related signaling pathways, such as type II diabetes mellitus and the insulin signaling pathways, and EX-4 could aggravate the effect of SDF-1 on PDLSCs biological roles by regulating primary immunodeficiency and tight junction signaling pathways. In addition, we confirmed that the SDF-1 and EX-4 combined application could enhance PDLSCs biological activity and promote PDLSCs osteogenic differentiation by regulating metabolism, biosynthesis and immune-related signaling pathways. Our current study lays a solid foundation for exploring the effects of SDF-1 and EX-4 synergistic application on periodontal tissue regeneration.

## Supplemental Information

10.7717/peerj.12091/supp-1 Supplemental Information 1 Bar plot of differentially expressed genes (DEGs) of PDLSCs Bar plot of differentially expressed genes (DEGs) of PDLSCs in SDF-1 *vs* NC, EX-4 *vs* NC, SDF-1+EX-4 *vs* NC, SDF-1+EX-4 *vs* EX-4, SDF-1+EX-4 *vs* SDF-1, and EX-4 *vs* SDF-1. The red histogram indicates the number of genes that were significantly upregulated and the blue histogram indicates the number of genes that were downregulated (*p*-adjusted value <0.05; FDR <0.05). The numbers of the DEGs are indicated at the top of every bar.

10.7717/peerj.12091/supp-2 Supplemental Information 2 Functional enrichment analysis of DEGs in different groups GO enrichment analysis of the DEGs in SDF-1 *vs* NC (A), EX-4 *vs* NC (C), and SDF-1+EX-4 *vs* NC groups (E). KEGG enrichment analysis of the DEGs in the SDF-1 *vs* NC (B), EX-4 *vs* NC (D), and SDF-1+EX-4 *vs* NC groups (F). The size of the symbol represents the gene counts enriched in the signaling pathways. The color indicates the degree of statistical significance (A, C, E). The different color bars represent the different enrichment methods of the KEGG catalog (B, D, F).

10.7717/peerj.12091/supp-3 Supplemental Information 3 UpSet plot analysis of the DEGs UpSet plot analysis of the DEGs among the SDF-1 *vs* NC, EX-4 *vs* NC, SDF-1+EX-4 *vs* NC, SDF-1+EX-4 *vs* EX-4, SDF-1+EX-4 *vs* SDF-1, and EX-4 *vs* SDF-1 groups.

10.7717/peerj.12091/supp-4 Supplemental Information 4 Short Time-series Expression Miner (STEM) analysis in PDLSCs Short Time-series Expression Miner (STEM) analysis in PDLSCs cocultured with SDF-1, EX-4 and SDF-1+EX-4 (mock infection was designated NC; the first node was the SDF-1 stimulated group, the second node was the EX-4 stimulated group, and the final node was the SDF-1+EX-4 combined stimulated group).

10.7717/peerj.12091/supp-5 Supplemental Information 5 Network constructed through the STRING database Network constructed through the STRING database based on the DEGs in SDF-1 *vs* NC (A), EX-4 *vs* NC (B), SDF-1+EX-4 *vs* NC (C), SDF-1+EX-4 *vs* Ex-4 (D), SDF-1+EX-4 *vs* SDF-1 (E), and EX-4 *vs* SDF-1 (F).

10.7717/peerj.12091/supp-6 Supplemental Information 6 Network constructed through the GeneMANIA database Network constructed through the GeneMANIA database based on the core DEGs in SDF-1+EX-4 *vs* NC (left panel) and the network based on the core genes generated by SDF-1+EX-4 (right). The genes in circles with white slash are the actual dynamic DEGs. The dynamic DEGs and predicted genes are interact based on physical interactions, coexpression, predications, colocalization, pathways, genetic interactions and shared protein domains.

10.7717/peerj.12091/supp-7 Supplemental Information 7 GO and KEGG functional enrichment analysis results based on DEGs GO and KEGG functional enrichment analysis results based on DEGs in SDF-1 *vs* NC, EX-4 *vs* NC, SDF-1+EX-4 *vs* NC, SDF-1+EX-4 *vs* EX-4, SDF-1+EX-4 *vs* SDF-1, and EX-4 *vs* SDF-1. Related to Fig. 2 and Fig. S2.

10.7717/peerj.12091/supp-8 Supplemental Information 8 Gene symbols of all DEGs generated in various compared groups Related to Fig. 3A.

10.7717/peerj.12091/supp-9 Supplemental Information 9 Gene symbols of all DEGs generated in various compared groups Related to Fig. S3.

10.7717/peerj.12091/supp-10 Supplemental Information 10 Functional enrichment analysis of the specific DEGs in each two compared groups Functional enrichment analysis of the specific DEGs in each two compared groups (SDF-1 *vs* NC, EX-4 *vs* NC, SDF-1+EX-4 *vs* NC, SDF-1+EX-4 *vs* SDF-1, EX-4 *vs* SDF-1). Related to Fig. 3B.

10.7717/peerj.12091/supp-11 Supplemental Information 11 Short Time-series Expression Miner (STEM) analysis in PDLSCs Short Time-series Expression Miner (STEM) analysis in PDLSCs cocultured with SDF-1, EX-4 and SDF-1+EX-4. Related to Fig. 4A.

10.7717/peerj.12091/supp-12 Supplemental Information 12 Heat map of the 25 upregulated DEGs generated by the SDF-1 and EX-4 combined application Related to Fig. 4B.

10.7717/peerj.12091/supp-13 Supplemental Information 13 GeneMANIA analysis network analysis results based on the 25 DEGs Related to Fig. 4C.

10.7717/peerj.12091/supp-14 Supplemental Information 14 Functional enrichment analysis of genes presented in the GeneMANIA network of Fig. 4C Related to Fig. 4D.

10.7717/peerj.12091/supp-15 Supplemental Information 15 GSEA enrichment based on all genes GSEA enrichment based on all genes in SDF-1 *vs* NC, EX-4 *vs* NC, SDF-1+EX-4 *vs* NC, SDF-1+EX-4 *vs* EX-4, and SDF-1+EX-4 *vs* SDF-1. Related to Fig. 6.

## Additional Information and Declarations

### Data Availability

The following information was supplied regarding data availability: The RNA sequence data are available at the NCBI Gene Expression Omnibus (GEO): GSE163354.

## References

[ref-1] anonymous (2019). The gene ontology resource: 20 years and still Going strong. Nucleic Acids Research.

[ref-2] Bae YK, Kim GH, Lee JC, Seo BM, Joo KM, Lee G, Nam H (2017). The significance of SDF-1α-CXCR4 axis in in vivo angiogenic ability of human periodontal ligament stem cells. Molecules and Cells.

[ref-3] Baek JM, Cheon YH, Kwak SC, Jun HY, Yoon KH, Lee MS, Kim JY (2018). Claudin 11 regulates bone homeostasis via bidirectional EphB4-EphrinB2 signaling. Experimental & Molecular Medicine.

[ref-4] Bakhtiarizadeh MR, Salehi A, Alamouti AA, Abdollahi-Arpanahi R, Salami SA (2019). Deep transcriptome analysis using RNA-Seq suggests novel insights into molecular aspects of fat-tail metabolism in sheep. Scientific Reports.

[ref-5] Bartold PM, Shi S, Gronthos S (2006). Stem cells and periodontal regeneration. Periodontology 2000.

[ref-6] Chu CY, Cha ST, Lin WC, Lu PH, Tan CT, Chang CC, Lin BR, Jee SH, Kuo ML (2009). Stromal cell-derived factor-1alpha (SDF-1alpha/CXCL12)-enhanced angiogenesis of human basal cell carcinoma cells involves ERK1/2-NF-kappaB/interleukin-6 pathway. Carcinogenesis.

[ref-7] Conway JR, Lex A, Gehlenborg N (2017). UpSetR: an R package for the visualization of intersecting sets and their properties. Bioinformatics.

[ref-8] Demircioğlu D, Cukuroglu E, Kindermans M, Nandi T, Calabrese C, Fonseca NA, Kahles A, Lehmann KV, Stegle O, Brazma A, Brooks NA, Rätsch G, Tan P, Göke J (2019). A pan-cancer transcriptome analysis reveals pervasive regulation through alternative promoters. Cell.

[ref-9] Deng B, Zhu W, Duan Y, Hu Y, Chen X, Song S, Yi Z, Song Y (2019). Exendin-4 promotes osteogenic differentiation of adipose-derived stem cells and facilitates bone repair. Molecular Medicine Reports.

[ref-10] Ding G, Liu Y, Wang W, Wei F, Liu D, Fan Z, An Y, Zhang C, Wang S (2010). Allogeneic periodontal ligament stem cell therapy for periodontitis in swine. Stem Cells.

[ref-11] Douglas JV, Bianco S, Edlund S, Engelhardt T, Filter M, Günther T, Hu KM, Nixon EJ, Sevilla NL, Swaid A, Kaufman JH (2019). STEM: an open source tool for disease modeling. Health Security.

[ref-12] Du L, Feng R, Ge S (2016). PTH/SDF-1α cotherapy promotes proliferation, migration and osteogenic differentiation of human periodontal ligament stem cells. Cell Proliferation.

[ref-13] Farkas MH, Au ED, Sousa ME, Pierce EA (2015). RNA-Seq: improving our understanding of retinal biology and disease. Cold Spring Harbor Perspectives in Medicine.

[ref-14] Feng Y, Su L, Zhong X, Guohong W, Xiao H, Li Y, Xiu L (2016). Exendin-4 promotes proliferation and differentiation of MC3T3-E1 osteoblasts by MAPKs activation. Journal of Molecular Endocrinology.

[ref-15] Franz M, Rodriguez H, Lopes C, Zuberi K, Montojo J, Bader GD, Morris Q (2018). GeneMANIA update 2018. Nucleic Acids Research.

[ref-16] Gao L, Li S, Li Y (2018). Exendin-4 promotes the osteogenic differentiation of osteoblasts via the Hedgehog/Gli1 signaling pathway. American Journal of Translational Research.

[ref-17] Hu L, Liu Y, Wang S (2018). Stem cell-based tooth and periodontal regeneration. Oral Diseases.

[ref-18] Kanehisa M, Goto S (2000). KEGG: kyoto encyclopedia of genes and genomes. Nucleic Acids Research.

[ref-19] Kim D, Langmead B, Salzberg SL (2015). HISAT: a fast spliced aligner with low memory requirements. Nature Methods.

[ref-20] Kimura Y, Komaki M, Iwasaki K, Sata M, Izumi Y, Morita I (2014). Recruitment of bone marrow-derived cells to periodontal tissue defects. Frontiers in Cell and Developmental Biology.

[ref-21] Lee JS, Jin Y, Park HJ, Yang K, Lee MS, Yang HS, Cho SW (2017a). In situ bone tissue engineering with an endogenous stem cell mobilizer and osteoinductive nanofibrous polymeric scaffolds. Biotechnology Journal.

[ref-22] Lee JS, Lee JB, Cha JK, Choi EY, Park SY, Cho KS, Kim CS (2017b). Chemokine in inflamed periodontal tissues activates healthy periodontal-ligament stem cell migration. Journal of Clinical Periodontology.

[ref-23] Li W (2012). Volcano plots in analyzing differential expressions with mRNA microarrays. Journal of Bioinformatics and Computational Biology.

[ref-24] Liang Q, Du L, Zhang R, Kang W, Ge S (2021). Stromal cell-derived factor-1/Exendin-4 cotherapy facilitates the proliferation, migration and osteogenic differentiation of human periodontal ligament stem cells in vitro and promotes periodontal bone regeneration in vivo. Cell Proliferation.

[ref-25] Liu H, Zheng J, Zheng T, Wang P (2019a). Exendin-4 regulates Wnt and NF- *κ*B signaling in lipopolysaccharide-induced human periodontal ligament stem cells to promote osteogenic differentiation. International Immunopharmacology.

[ref-26] Liu J, Ruan J, Weir MD, Ren K, Schneider A, Wang P, Oates TW, Chang X, Xu HHK (2019b). Periodontal bone-ligament-cementum regeneration via scaffolds and stem cells. Cell.

[ref-27] Liu Y, Zheng Y, Ding G, Fang D, Zhang C, Bartold PM, Gronthos S, Shi S, Wang S (2008). Periodontal ligament stem cell-mediated treatment for periodontitis in miniature swine. Stem Cells.

[ref-28] Luciani P, Fibbi B, Mazzanti B, Deledda C, Ballerini L, Aldinucci A, Benvenuti S, Saccardi R, Peri A (2018). The effects of Exendin-4 on bone marrow-derived mesenchymal cells. Endocrine.

[ref-29] Meng J, Ma X, Wang N, Jia M, Bi L, Wang Y, Li M, Zhang H, Xue X, Hou Z (2016). Activation of GLP-1 receptor promotes bone marrow stromal cell osteogenic differentiation through *β*-Catenin. Stem Cell Reports.

[ref-30] Nguyen TS, Lapidot T, Ruf W (2018). Extravascular coagulation in hematopoietic stem and progenitor cell regulation. Blood.

[ref-31] Nilsson M, Holst JJ, Björck IM (2007). Metabolic effects of amino acid mixtures and whey protein in healthy subjects: studies using glucose-equivalent drinks. The American Journal of Clinical Nutrition.

[ref-32] Onizuka S, Iwata T (2019). Application of periodontal ligament-derived multipotent mesenchymal stromal cell sheets for periodontal regeneration. International Journal of Molecular Sciences.

[ref-33] Ozsolak F, Milos PM (2011). RNA sequencing: advances, challenges and opportunities. Nature Reviews. Genetics.

[ref-34] Peres MA, Macpherson LMD, Weyant RJ, Daly B, Venturelli R, Mathur MR, Listl S, Celeste RK, Guarnizo-Herreño CC, Kearns C, Benzian H, Paul Allison, Watt RG (2019). Oral diseases: a global public health challenge. Lancet.

[ref-35] Pertea M, Pertea GM, Antonescu CM, Chang TC, Mendell JT, Salzberg SL (2015). StringTie enables improved reconstruction of a transcriptome from RNA-seq reads. Nature Biotechnology.

[ref-36] Ranganathan K, Agarwal S, Cholok D, Loder S, Li J, Sung Hsieh HH, Wang SC, Buchman SR, Levi B (2016). The role of the adaptive immune system in burn-induced heterotopic ossification and mesenchymal cell osteogenic differentiation. The Journal of Surgical Research.

[ref-37] Singh S, Wright Jr EE, Kwan AY, Thompson JC, Syed IA, Korol EE, Waser NA, Yu MB, Juneja R (2017). Glucagon-like peptide-1 receptor agonists compared with basal insulins for the treatment of type 2 diabetes mellitus: a systematic review and meta-analysis. Diabetes, Obesity & Metabolism.

[ref-38] Subramanian A, Tamayo P, Mootha VK, Mukherjee S, Ebert BL, Gillette MA, Paulovich A, Pomeroy SL, Golub TR, Lander ES, Mesirov JP (2005). Gene set enrichment analysis: a knowledge-based approach for interpreting genome-wide expression profiles. Proceedings of the National Academy of Sciences of the United States of America.

[ref-39] Wang F, Du L, Ge S (2016). PTH/SDF-1α cotherapy induces CD90+CD34- stromal cells migration and promotes tissue regeneration in a rat periodontal defect model. Scientific Reports.

[ref-40] Wang L, Wang S, Li W (2012). RSeQC: quality control of RNA-seq experiments. Bioinformatics.

[ref-41] Wang Y, Emeto TI, Lee J, Marshman L, Moran C, Seto SW, Golledge J (2015). Mouse models of intracranial aneurysm. Brain Pathology.

[ref-42] Wang Y, Wang Q, Arora PD, Rajshankar D, McCulloch CA (2013). Cell adhesion proteins: roles in periodontal physiology and discovery by proteomics. Periodontology 2000.

[ref-43] Yang Y, Zhou Y, Wang Y, Wei X, Wu L, Wang T, Ma A (2020). Exendin-4 reverses high glucose-induced endothelial progenitor cell dysfunction via SDF-1β/CXCR7-AMPK/p38-MAPK/IL-6 axis. Acta Diabetologica.

[ref-44] Yap MKK, Misuan N (2019). Exendin-4 from Heloderma suspectum venom: from discovery to its latest application as type II diabetes combatant. Basic & Clinical Pharmacology & Toxicology.

[ref-45] Zhang Y, Chen S, Liu B, Zhou H, Hu S, Zhou Y, Han T, Chen Y (2016). Exendin-4 promotes proliferation of adipose-derived stem cells through ERK and JNK signaling pathways. In Vitro Cellular & Developmental Biology. Animal.

[ref-46] Zhou H, Li D, Shi C, Xin T, Yang J, Zhou Y, Hu S, Tian F, Wang J, Chen Y (2015a). Effects of Exendin-4 on bone marrow mesenchymal stem cell proliferation, migration and apoptosis in vitro. Scientific Reports.

[ref-47] Zhou H, Yang J, Xin T, Zhang T, Hu S, Zhou S, Chen G, Chen Y (2015b). Exendin-4 enhances the migration of adipose-derived stem cells to neonatal rat ventricular cardiomyocyte-derived conditioned medium via the phosphoinositide 3-kinase/Akt-stromal cell-derived factor-1α/CXC chemokine receptor 4 pathway. Molecular Medicine Reports.

[ref-48] Zhou Y, Zhou B, Pache L, Chang M, Khodabakhshi AH, Tanaseichuk O, Benner C, Chanda SK (2019). Metascape provides a biologist-oriented resource for the analysis of systems-level datasets. Nature Communications.

[ref-49] Zhu L, Dissanayaka WL, Zhang C (2019). Dental pulp stem cells overexpressing stromal-derived factor-1α and vascular endothelial growth factor in dental pulp regeneration. Clinical Oral Investigations.

