# Supplementary figures and images for: Transcriptome analysis reveals the mechanism of stromal cell-derived factor-1 and exendin-4 synergistically promoted periodontal ligament stem cells osteogenic differentiation
Source: PeerJ. 2021 Aug 27;9:e12091. doi: 10.7717/peerj.12091 (PMC8404574; doi:10.7717/peerj.12091)

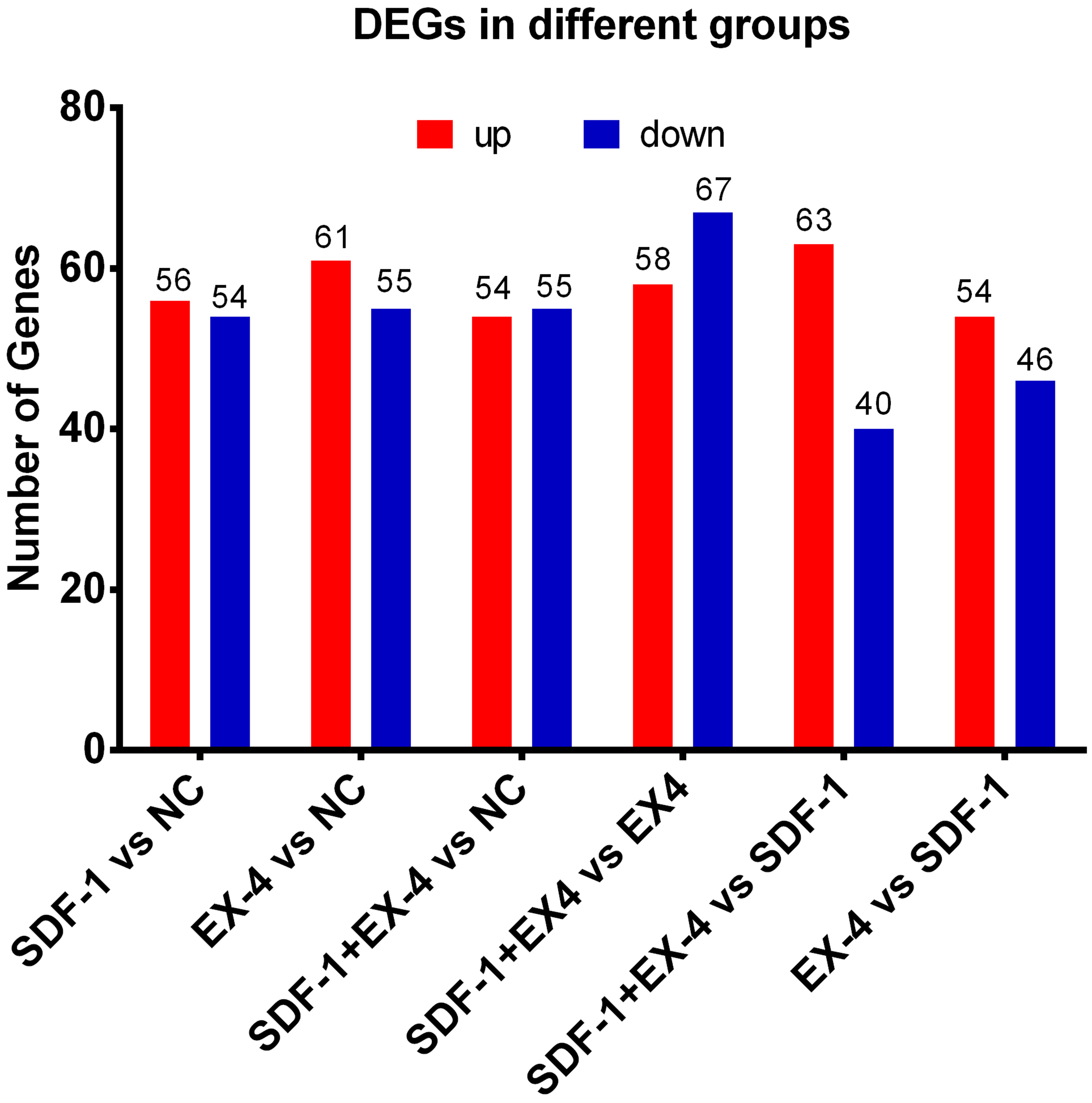

Supplement: Supplemental Information 1 — Bar plot of differentially expressed genes (DEGs) of PDLSCs in SDF-1 vs NC, EX-4 vs NC, SDF-1+EX-4 vs NC, SDF-1+EX-4 vs EX-4, SDF-1+EX-4 vs SDF-1, and EX-4 vs SDF-1. The red histogram indicates the number of genes that were significantly upregulated and the blue histogram indicates the number of genes that were downregulated (p-adjusted value <0.05; FDR <0.05). The numbers of the DEGs are indicated at the top of every bar. [file peerj-09-12091-s001.png]

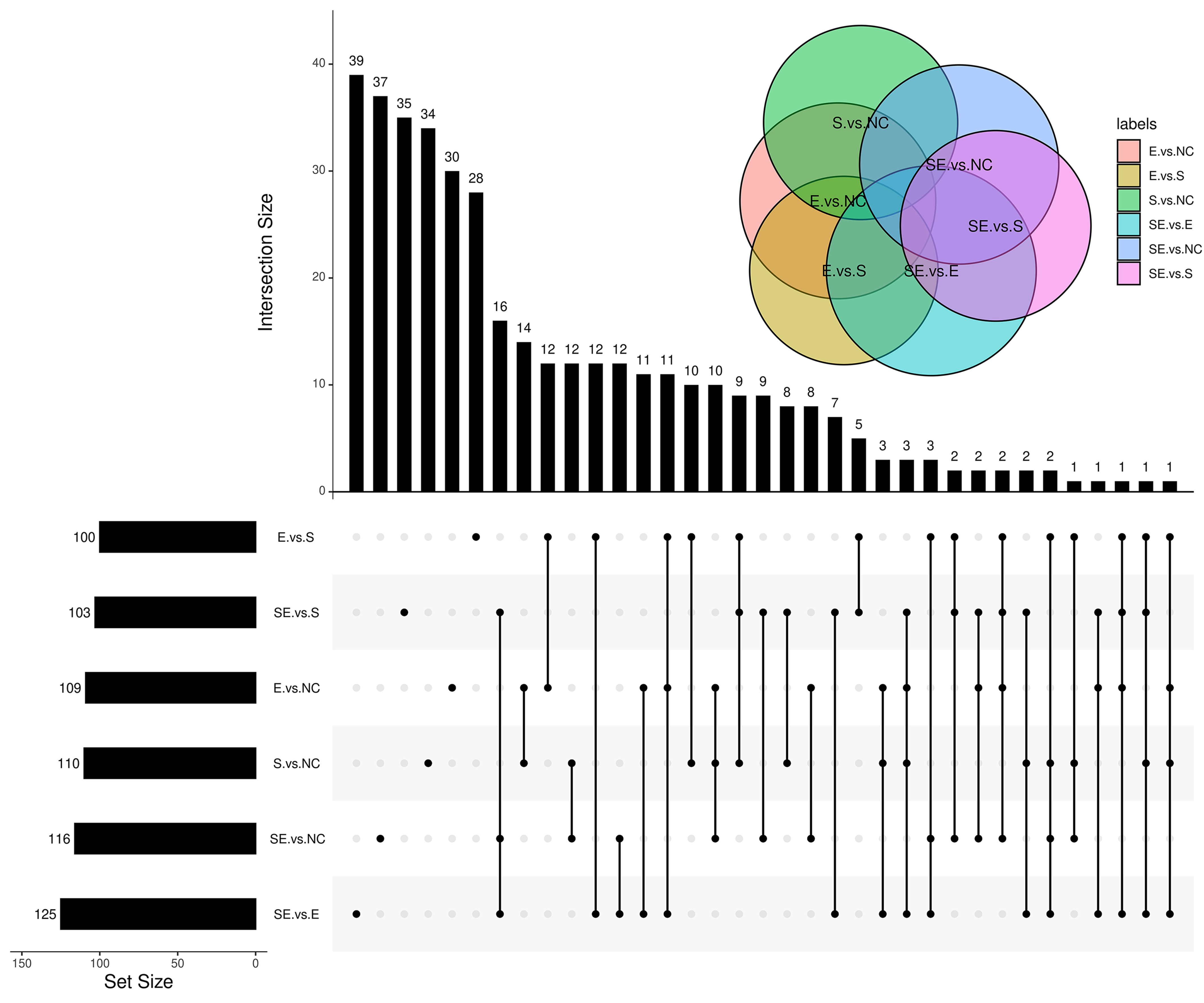

Supplement: Supplemental Information 3 — UpSet plot analysis of the DEGs among the SDF-1 vs NC, EX-4 vs NC, SDF-1+EX-4 vs NC, SDF-1+EX-4 vs EX-4, SDF-1+EX-4 vs SDF-1, and EX-4 vs SDF-1 groups. [file peerj-09-12091-s003.png]

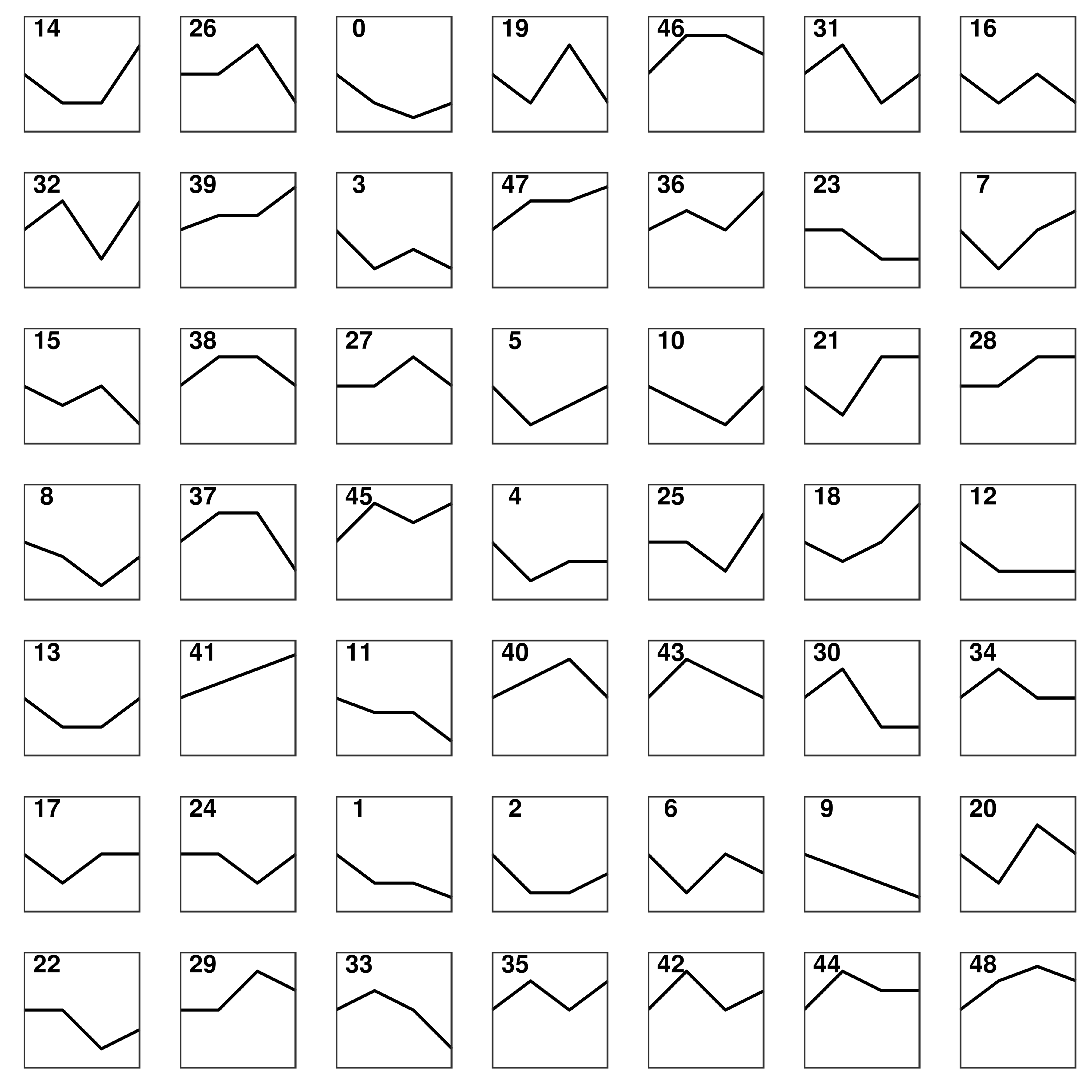

Supplement: Supplemental Information 4 — Short Time-series Expression Miner (STEM) analysis in PDLSCs cocultured with SDF-1, EX-4 and SDF-1+EX-4 (mock infection was designated NC; the first node was the SDF-1 stimulated group, the second node was the EX-4 stimulated group, and the final node was the SDF-1+EX-4 combined stimulated group). [file peerj-09-12091-s004.png]

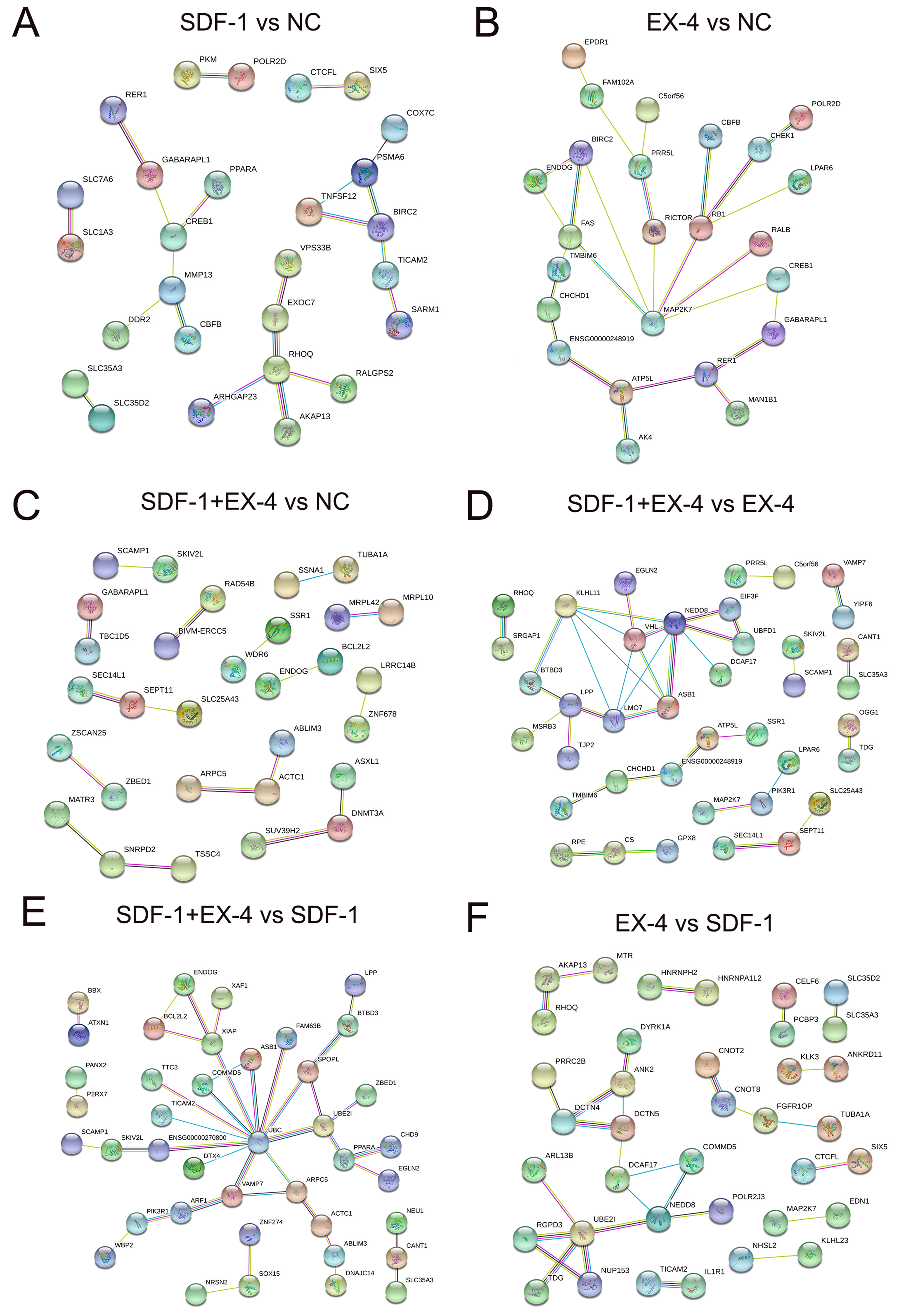

Supplement: Supplemental Information 5 — Network constructed through the STRING database based on the DEGs in SDF-1 vs NC (A), EX-4 vs NC (B), SDF-1+EX-4 vs NC (C), SDF-1+EX-4 vs Ex-4 (D), SDF-1+EX-4 vs SDF-1 (E), and EX-4 vs SDF-1 (F). [file peerj-09-12091-s005.png]

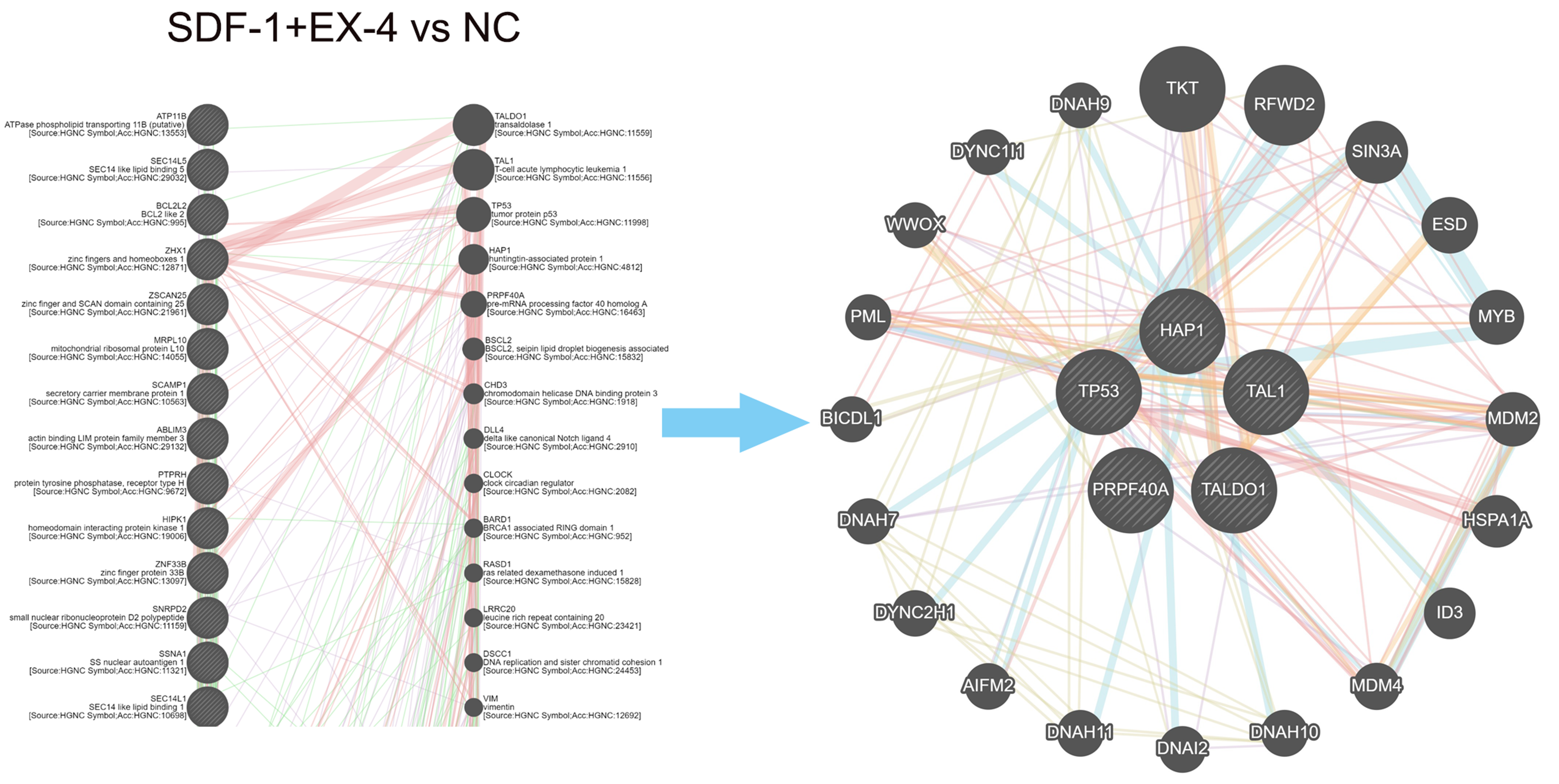

Supplement: Supplemental Information 6 — Network constructed through the GeneMANIA database based on the core DEGs in SDF-1+EX-4 vs NC (left panel) and the network based on the core genes generated by SDF-1+EX-4 (right). The genes in circles with white slash are the actual dynamic DEGs. The dynamic DEGs and predicted genes are interact based on physical interactions, coexpression, predications, colocalization, pathways, genetic interactions and shared protein domains. [file peerj-09-12091-s006.png]
